# TLR2 and TLR4 Surface and Gene Expression in White Blood Cells after Fasting and Oral Glucose, Lipid and Protein Challenges: Influence of Obesity and Sex Hormones

**DOI:** 10.3390/biom10010111

**Published:** 2020-01-09

**Authors:** M. Ángeles Martínez-García, Miriam Ojeda-Ojeda, Eulalia Rodríguez-Martín, María Insenser, Samuel Moncayo, Francisco Álvarez-Blasco, Manuel Luque-Ramírez, Héctor F. Escobar-Morreale

**Affiliations:** 1 Diabetes, Obesity and Human Reproduction Research Group, Department of Endocrinology and Nutrition, Hospital Universitario Ramón y Cajal &Universidad de Alcalá &Instituto Ramón y Cajal de Investigación Sanitaria (IRYCIS) & Centro de Investigación Biomédica en Red Diabetes y Enfermedades Metabólicas Asociadas (CIBERDEM), 28034 Madrid, Spain; manitamg@yahoo.es (M.Á.M.-G.); ojedaojedamiriam@gmail.com (M.O.-O.); mariarosa.insenser@salud.madrid.org (M.I.); samuel.moncayo@gmail.com (S.M.); fablas74@gmail.com (F.Á.-B.); manuel.luque@salud.madrid.org (M.L.-R.); 2 Department of Immunology, Hospital Universitario Ramón y Cajal, 28034 Madrid, Spain; eulalia.rodriguez@salud.madrid.org

**Keywords:** sex hormones, leukocytes, macronutrient loads, obesity, polycystic ovary syndrome, toll-like receptors, postprandial response

## Abstract

We studied if macronutrients of the diet have different effects on leukocyte activation, and if these effects are influenced by sex hormones or obesity. We analyzed leukocyte cell surface and gene expression of toll-like receptors 2 and 4 (TLR2 and TLR4) during fasting and after macronutrient loads in women with polycystic ovary syndrome and female and male controls. Fasting TLR2 surface expression in neutrophils was higher in men than in women. Obese subjects presented higher *TLR2* gene expression than nonobese individuals, particularly in men. In contrast, surface TLR4 expression was lower in men and in obese individuals. Postprandial cell-surface expression decreased similarly after all macronutrient loads. Neutrophil TLR2 decreased only in obese subjects whereas TLR4 showed a greater decrease in nonobese individuals. However, *TLR2* gene expression increased after glucose ingestion and decreased during the lipid load, while *TLR4* was induced in response to lipids and mostly to glucose. Postprandial TLR gene expression was not influenced by group of subjects or obesity. Both cell-surface and gene postprandial expression inversely correlated with their fasting levels. These responses suggest a transient compensatory response aiming to prevent postprandial inflammation. However, obesity and sex hormones showed opposite influences on surface expression of TLR2 and TLR4, but not on their gene expression, pointing to regulatory posttranscriptional mechanisms.

## 1. Introduction

Pathogen and nutrient response pathways are evolutionarily conserved and highly integrated to regulate metabolic and immune homeostasis. Metabolic and inflammatory pathways converge at different levels, including that of cell-surface receptors such as toll-like receptors (TLRs). TLRs comprise a family of proteins that recognizes pathogen-associated molecular patterns (PAMPs) and initiates host innate immune responses. These molecular sites allow for the coordination between nutrient-sensing and immune response pathways in order to maintain homeostasis under diverse metabolic and immune conditions [1,2]. Among the TLR family members, TLR2 and TLR4 are considered important regulators of metabolic inflammation during the development of obesity and related comorbidities [2]. The activation of TLR signaling requires ligand binding and recruitment and internalization of adaptor molecules [3]. However, other types of accessory molecules also modulate TLR responses including the scavenger receptor CD36 [4,5] and the costimulatory molecule CD86 [6,7]. Excessive nutrients can be sensed by these innate pattern recognition receptors as danger signals, either directly or through production of endogenous ligands or through the modulation of intestinal microbiota, triggering the activation of downstream inflammatory cascades and ending with the production of inflammatory cytokines and immune cell infiltration in metabolic tissues [3].

The inflammatory response can be damaging to the host if not regulated properly. In agreement, certain disorders such as obesity and type 2 diabetes—characterized by the existence of a chronic low-grade inflammatory state [8]—have been associated with increased TLR2/4 activation [9,10,11]. In addition, evidence derived mostly from the study of the androgen excess disorder polycystic ovary syndrome (PCOS) suggests that sex hormones play a major role in the pathogenesis of obesity and related metabolic comorbidities [12]. In fact, PCOS is considered a low-grade inflammatory condition and there is evidence that hyperandrogenism is linked to inflammation [13,14].

Interactions between sex hormones and the immune system with consistent sexdisparities in immunity have been known for a long time [15,16]. Males and females differ in their innate and adaptive immune responses to foreign and self-antigens, in the susceptibility to infection by pathogens and viruses and in the prevalence of autoimmune diseases, this latter being increased in women as a result of the higher activity of its immune system [16]. In addition to the X-linked genetic component, this sexual dimorphism is mainly due to the differential impact of sex steroids (mostly estrogen but also progesterone and testosterone) on the activity of immune cells through the control of cytokine and immunoglobulin production, as well as of TLR expression [17,18,19].

A rise in inflammation also takes place acutely following meals and several cells, including immune cells, responds to the postprandial elevation of several meal components by mounting transient hormonal, metabolic, inflammatory, and oxidative responses [20,21,22,23]. Diet composition, and not only the amount of food ingested, exerts a direct impact on postprandial inflammation. Different influences on inflammatory markers have been demonstrated for carbohydrates, fibers, and fat [21,24], and these influences are not restricted to long-term effects but also occur in the immediate postprandial period [23,25,26]. Previous studies have evaluated the in vitro effects of hyperglycemic conditions and free fatty acid exposition in cells from diverse origin [27]. However, literature regarding the separate influences of isolated macronutrient loads in vivo, particularly proteins, is relatively scarce [28,29,30,31,32].

In order to provide new insights on the role of TLRs in the integration of metabolic and immune responses at fasting and during single macronutrient loads, while considering the possible influences of sexual hormones and body weight on these responses, we evaluated the expression patterns of TLR2 and TLR4 in peripheral blood leukocytes after oral glucose, lipid, and protein challenges in a group of young adults, including control women, women with PCOS, and men.

## 2. Materials and Methods

### 2.1. Subjects

We included 53 young adults: Seventeen women with PCOS, 17 non-hyperandrogenic control women and 19 control men. Female and male controls were selected so that they were similar in terms of age and body mass index (BMI). Subjects were classified into non-obese (BMI < 30 kg/m^2^) or obese (BMI ≥ 30 kg/m^2^) subgroups. The diagnosis of PCOS was based on the presence of clinical and/or biochemical hyperandrogenism, oligo-ovulation, and exclusion of secondary etiologies [33]. The control groups were composed of patients reporting because of weight excess and of healthy non-obese volunteers. No women in the control group had signs or symptoms of hyperandrogenism, history of menstrual dysfunction, or infertility. Before enrolment, the participants had no history of obesity-associated comorbidities including disorders of glucose tolerance, hypertension, cardiovascular disease, or sleep apnea. The subjects had not received treatment with oral contraceptives, antiandrogens, insulin sensitizers, statins, antihypertensives, or drugs that might interfere with clinical and/or biochemical variables or influence body fat depots for at least six months prior to the study.

### 2.2. Ethics

The study (PI11/00357) was approved by the Ethics Committee of the Hospital Universitario Ramón y Cajal on 4 November 2011. We obtained written informed consent from all participants.

### 2.3. Study Design

All individuals underwent a comprehensive clinical, anthropometric, and physical evaluation. Patients were instructed to follow a diet unrestricted in carbohydrates (at least 300 g of carbohydrates per day during three days) before sampling in order to avoid false positive results in the 75 g oral glucose tolerance test, which was used not only for research purposes but also to check the patients for disorders of glucose tolerance. We obtained serum and plasma samples after a 12 h overnight fasting, and during the follicular phase of the menstrual cycle or in amenorrhea after excluding pregnancy in women.

On alternate days, we submitted patients to separate oral loads in the following order: glucose, lipids, and proteins. The protocol for macronutrient challenges has been described elsewhere [34]. Challenges could not be randomized because the oral glucose tolerance test was also used for the diagnosis of diabetes and had to be conducted after the three-day diet; hence, the glucose challenge needed to be the first oral load to be conducted in all the subjects. Quantities and volumes in the oral loads were adjusted for a total caloric intake of 300 Kcal. Therefore, we administered 200 mL of a 37.5 g/dL glucose solution (GlycoSullNaranja 75 g, QuímicaClínicaAplicada, Spain) for the oral glucose tolerance test, 66 mL of a 4.5 kcal/mL long-chain trigycerides enteral nutrition supplement (Supracalneutro, Nutricia S.R.L., Spain) for the oral lipid load, and 75 g of an enteral nutrition supplement containing caseinates (Proteína NM, NutriciónMédica S.L., Spain) for the oral protein challenge. Postprandial samples were obtained at 60 and 120 min after the glucose and protein loads, and after 120 and 240 min during the lipid challenge, considering the slower intestinal absorption rate of lipids [35]. Additional information about the oral challenges is available in Supplementary Methods.

Technical characteristics of the assays used for laboratory measurements have been described in detail elsewhere [36]. Serum concentrations of high-sensitivity C-reactive protein (hsCRP) were measured by commercial immuno-chemiluminescence (Immulite 2000 high-sensitivity CRP, Siemens Healthcare, Los Angeles, CA, USA). The composite insulin sensitivity index (ISI) was calculated from the circulating glucose and insulin concentrations during the oral glucose tolerance test [37]. Homeostasis model assessment of insulin resistance (HOMA-IR) was calculated from fasting glucose and insulin concentrations [38].

### 2.4. Flow Cytometry

The cell-surface expression of TLR2 and TLR4 on monocytes and neutrophils, and of leukocyte activation markers CD36 and CD86 on monocytes, was detected by direct immunofluorescence and quantified by flow cytometry. Fresh blood samples were collected during fasting and 2h (glucose and proteins) or 4h (lipids) after the oral challenges and immediately assayed by a three-color flow cytometry. Briefly, 100 µL aliquots of whole blood were stained according to the manufacturer´s instructions (BD Biosciences, San Jose, CA, USA) for 25 min in the dark at room temperature with the following directly conjugated fluorescent-labeled monoclonal antibodies (mAbs) as appropriate: CD282 (TLR2)-fluorescein isothiocyanate (FITC) (clone TL2.1, eBioscience, San Diego, CA, USA), CD284 (TLR4)-phycoerythrin (PE) (clone HTA125, eBioscience), CD36-PE (clone CB38, BD Biosciences), CD86-FITC (clone 2331 FUN-1, BD Biosciences), CD14-FITC (clone M5E2, BD Biosciences), CD33-PE (clone WM53, BD Biosciences), and CD45-peridinin–chlorophyll protein (PerCP) (clone 2D1, BD Biosciences). The corresponding isotypecontrols were used to detect nonspecific staining. Erythrocytes were lysed with FACS lysing solution (BD Biosciences) for 10 min at room temperature. The immuno-stained cells were washed two times with PBS and the remaining leukocytes were analyzed in a fluorescence-activated cell counter (BD FACSCalibur, BD Biosciences). Cytometry data analysis was performed using the FCS Express 4 software package (De Novo software, Glendale, CA, USA). Ten thousand events were acquired, and neutrophils and monocytes were identified and gated according to their characteristic CD45+ staining and side-scatter profiles, excluding cellular debris (Figure S1). In addition, a combination of CD14 or CD33 with TLR2/TLR4 or CD36/CD86 was also used to improve monocyte and neutrophil identification. Mean fluorescence intensity (MFI) derived from fluorescence histogram was used to study cell-surface expression levels of TLR2, TLR4, and leukocyte activation markers. Results were expressed as the ratio of the MFI of the mAb of interest to the MFI of the matched isotypic negative control, with the aim of correcting for nonspecific antibody binding. For technical reasons, flow cytometry analyses were possible for fasting samples from 44 individuals and for macronutrient responses of 37 individuals.

### 2.5. Gene Expression

We performed gene expression studies to evaluate the relative quantification of *TLR2* and *TLR4* genes in peripheral blood leukocytes by quantitative real-time PCR experiments (qPCR) as recently described [39]. Additional information about these procedures is available in Supplementary Methods. Leukocytes were immediately isolated after fasting and during the postprandial phase (60 and 120 min after the glucose and protein loads, or 120 and 240 min during the lipid challenge). Total RNA was extracted from frozen isolated leukocytes preserved in RNA later by using the Qiagen miRN easy mini kit including an RNase-free DNase treatment (QIAGEN, Hilden, Germany). RNA yield, quality, and purity were assessed with a NanoDrop2000 spectrophotometer and the Qubit RNA HS assay kit in a Qubit3.0 fluorimeter (Life Technologies-Invitrogen). RNA integrity number was evaluated with the high sensitivity RNA Screen Tape in a TapeStation2200 (Agilent Technologies). All the samples evaluated presented RNA integrity number ≥ 8.5. Equal amounts of RNA from all samples were reverse-transcribed with the RT2 First Strand cDNA synthesis kit (QIAGEN). *RPS18* and *HPRT1* among seven genes evaluated were found to be more consistent in their expression and therefore served as reference for data normalization. Experiments were performed in predesigned Human CustomSignArrays384 qPCR panels with Perfect Master Mix SYBR Green (Any Genes, France) on a LightCycler480 instrument, software version 1.5 (ROCHE). Data were expressed as arbitrary units using the log2^−ΔCq^ transformation. All samples were assayed in duplicate and negative and positive controls were included in each plate.

### 2.6. Sample Size Analysis

We used the online sample size and power calculator provided by the Institut Municipal d’InvestigacióMèdica from Barcelona, Spain, version 7.12 (https://www.imim.cat/ofertadeserveis/software-public/granmo/, last accessed 7 February 2019). This calculation was based on previous results of Gonzalez et al. [40] reporting differences between women with PCOS and controls in the percentage change of nuclear factor kappaB expression in mononuclear cells after a standard OGTT. Setting alpha at 0.05 and beta at 0.2 for a two-sided test, the inclusion of eight patients per group would allow to recognize as statistically significant a mean difference of 50.35% change, assuming a standard deviation of 34.1% change.

### 2.7. Statistical Analysis

Data are mean ± SD (tables) or mean ± SEM (figures). Logarithmic transformations were applied as needed to ensure normal distribution of data. Univariate general linear models (GLMs) were used to determine within a single analysis the influence of group (i.e., control women, women with PCOS, and men), obesity and the interaction of both factors on hormonal, metabolic, and inflammatory variables at fasting, while adjusting the level of significance to compensate for the multiple comparisons involved. The mean of the three measurements obtained at fasting—before each macronutrient load—were used for these analyses. To evaluate the response to macronutrient ingestion of cell-surface and gene expression and the influence of group and obesity or a possible interaction of both variables on these responses we used repeated-measures GLMs in two distinct analyses. We introduced as within-subjects factor: (i) Fasting and postprandial values to evaluate significant changes from fasting; and (ii) the responses to the separate oral challenges expressed as areas under the curve (AUC) to evaluate differences among macronutrients, also introducing group and obesity as between-subjects factors. For each dependent variable, the AUC was calculated using the trapezoidal rule. The AUC was subsequently corrected for fasting levels—to comprise only the net increment or decrement in the dependent variable—and was normalized by the whole duration of the challenge to warrant comparison among macronutrient loads (120 min for glucose and protein loads and 240 min for lipid load). The Mauchly’s test was used to estimate sphericity and the Greenhouse–Geisser correction factor was applied as needed. In all the analyses, Fisher’s Least Significant Difference post-hoc test was used for multiple comparisons among groups. The relationships between continuous variables were assessed by Pearson’s or Spearman’s correlation analysis as needed. All the analyses were performed with SPSS Statistics 15.0 (SPSS Inc., Chicago, IL, USA) and *p*-values < 0.05 were considered statistically significant.

## 3. Results

### 3.1. Effect of Group and Obesity on Clinical, Hormonal, and Biochemical Variables

Fasting clinical, hormonal, and metabolic characteristics of participants are shown in Table S1. Men presented higher waist circumference and waist-to-hip ratio but lower sex hormone-binding globulin (SHBG) and high density-lipoprotein (HDL) cholesterol compared with both groups of women. In addition to the expected highest total and free testosterone concentrations in men, women with PCOS also presented increased total and free testosterone concentrations compared with control women. Free testosterone/free estradiol ratio was higher in men and in PCOS women compared with controls. Accordingly, control women and PCOS patients had higher total and free estradiol levels than men. The hirsutism score was only assessed in women and was increased in PCOS patients compared with controls. There were no differences in insulin resistance according to the ISI or HOMA-IR scores among groups. Obese subjects presented higher waist circumference and waist-to-hip ratio, free testosterone, total and free estradiol, hsCRP, fasting insulin and glucose, HOMA-IR and low density-lipoprotein (LDL) cholesterol levels, and lower SHBG, ISI and HDL-cholesterol concentrations than non-obese individuals. We did not observe any significant interaction between group of subjects and obesity on these variables.

### 3.2. Influence of Group and Obesity on Cell-Surface and Gene Expression of TLR2, TLR4, and on Cell-Surface Expression of CD36 and CD86 at Fasting

Cell-surface constitutive expression was observed for all leukocyte activation markers after an overnight fasting (Table 1, Tables S2 and S3). Irrespective of weight status, TLR2 surface expression in neutrophils was higher in men compared with both groups of women, and tended to be higher in obese subjects compared with non-obese individuals (Table 1, *p* = 0.077). Accordingly, *TLR2* gene expression was greater in obese subjects compared with non-obese participants (Table 1, *p* = 0.034), this effect tending to be driven by the group of men (Table 1, *p* = 0.072). On the contrary, TLR2 surface expression in monocytes was unaffected by group of subjects and obesity (Table 1).

In contrast, TLR4 expression in both monocytes and neutrophils was lower in men compared with PCOS women, and was also decreased in obese subjects compared with non-obese individuals regardless of the group of subjects (Table 1). However, fasting *TLR4* gene expression levels showed no differences in group, obesity, or in their interaction (Table 1).

Monocyte CD36 and CD86 surface expression showed higher levels in non-obese compared with obese participants (Table 1). Of note, the expression of TLR2 in neutrophils correlated negatively with that of TLR4 and CD36 in monocytes. Furthermore, TLR4 levels both in monocytes and neutrophils strongly positively correlated with CD36 while TLR2 in monocytes did the same with CD86. These correlations and those between fasting expression of monocyte and neutrophil surface markers with clinical, hormonal, and metabolic parameters are summarized in Table S4. Finally, expression of both TLR genes did not correlate with their corresponding surface levels neither in monocytes nor in neutrophils (data not shown).

### 3.3. Effect of Macronutrient Challenges on Cell-Surface and Gene Expression of TLR2, TLR4, and on Cell-Surface Expression of CD36 and CD86

**Figure 1:**
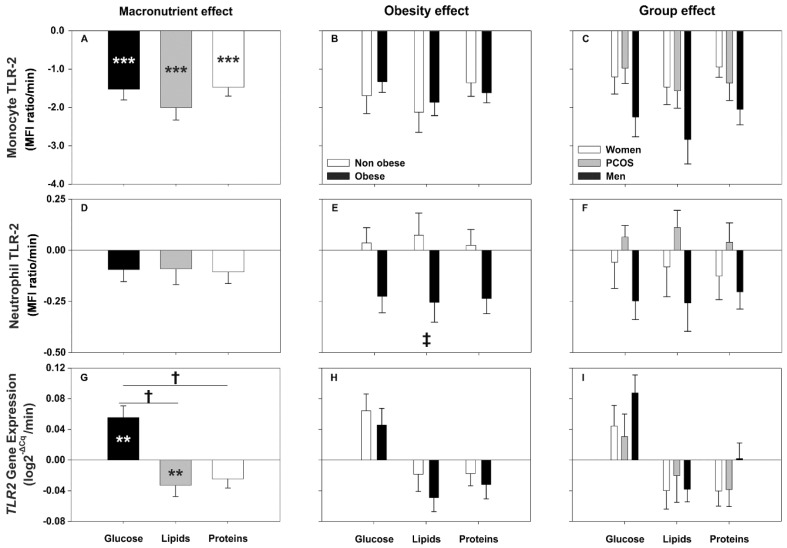
Areas under the curve (AUC) of TLR2 surface and gene expression in response to macronutrient challenges and according to obesity and group. Data are means ± SEM. *p*-values represent the differences within and between macronutrient loads as analyzed by repeated-measures general linear models (GLM) (introducing fasting and postprandial levels or the AUC of each load, respectively, as within-subjects effect, and obesity and group as between-subjects effects). ** *p* < 0.01 and *** *p* < 0.001 for differences from fasting levels within macronutrient loads. † *p* < 0.001 for differences among macronutrients, irrespective of obesity and group. ‡ *p* < 0.05 for differences in obesity, irrespective of macronutrient and group.

**Figure 2:**
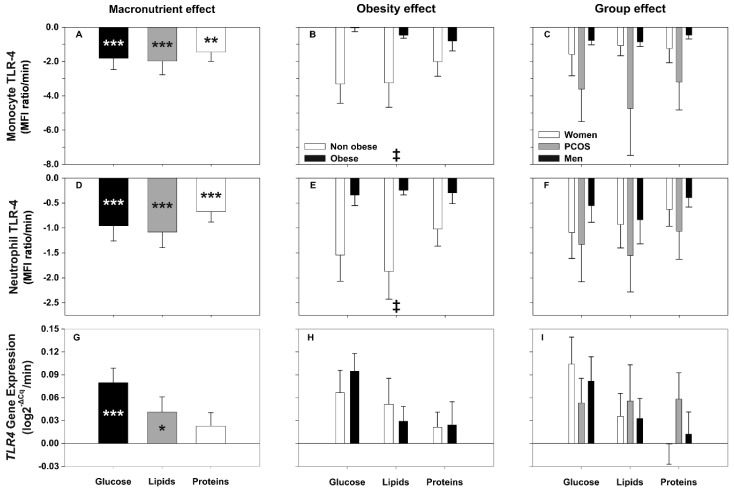
Areas under the curve (AUC) of TLR4 surface and gene expression in response to macronutrient challenges and according to obesity and group. Data are means ± SEM. *p*-values represent the differences within and between macronutrient loads analyzed by repeated-measures GLM (introducing fasting and postprandial levels or the AUC of each load, respectively, as within-subjects effect, and obesity and group as between-subjects effects). * *p* < 0.05, ** *p* < 0.01 and *** *p* < 0.001 for differences from fasting levels within macronutrient loads. ‡ *p* < 0.05 for differences in obesity, irrespective of macronutrient and group.

**Figure 3:**
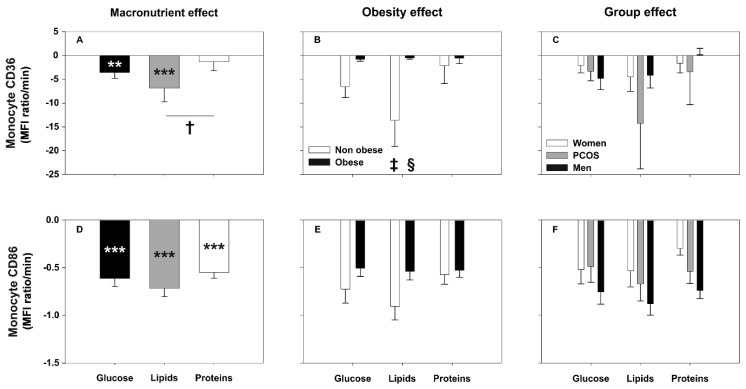
Areas under the curve (AUC) of monocyte CD36 and CD86 surface expression in response to macronutrient challenges and according to obesity and group. Data are means ± SEM. *p*-values represent the differences within and between macronutrient loads analyzed by repeated-measures GLM (introducing fasting and postprandial levels or the AUC of each load, respectively, as within-subjects effect, and obesity and group as between-subjects effects). ** *p* < 0.01 and *** *p* < 0.001 for differences from fasting levels within macronutrient loads. † *p* < 0.05 for differences among macronutrients, irrespective of obesity and group. ‡ *p* < 0.05 for differences in obesity, irrespective of macronutrient and group. § *p* < 0.05 for the interaction of obesity with macronutrients.

Regardless of the macronutrient being administered, the postprandial period was characterized by a general decrease in the cell-surface expression of the markers studied here (Figure 1A, Figure 2A,D and Figure 3A,D; Tables S2 and S3). When we compared the AUCs of each marker among the distinct macronutrient loads we did not find significant differences between them, either on monocytes or neutrophils (Figure 1A,D, Figure 2A,D and Figure 3D). Nevertheless, we observed a general trend towards a smaller decrease after the protein load compared with those observed after lipid ingestion that reached statistical significance for CD36 (Figure 3A). Of note, the AUCs of most of the markers showed strong inverse correlations with their respective fasting levels, especially for TLR4. Similarly, the AUC of both *TLR2* and *TLR4* gene expression negatively correlated with its fasting values, especially during the lipid and glucose challenges (Table 2).

#### 3.3.1. Effect of Group and Obesity on TLR2 Expression after Macronutrient Challenges

Surface expression of TLR2 in monocytes decreased significantly after the administration of all the macronutrients (Table S2), with a tendency (*p* = 0.081) towards a larger decrease after lipid ingestion compared with those observed after the intake of glucose and proteins (Figure 1A). Obesity did not influence such responses (Figure 1B and Table S2). Only the decrease in monocyte-TLR2 expression tended (*p* = 0.096) to be larger in men compared with control women irrespective of macronutrient loads (Figure 1C).

Surface expression of TLR2 in neutrophils did not change with respect to fasting levels regardless of the macronutrient administered (Figure 1D and Table S2). However, neutrophil TLR2 surface expression decreased in obese subjects and did not change in non-obese individuals (Figure 1E and Table S2), and tended (*p* = 0.074) to decrease in male controls compared with PCOS women (Figure 1F), independently of the macronutrient ingested.

*TLR2* gene expression increased after glucose ingestion but decreased after lipid and protein intake (Figure 1G), with the decrease observed after protein ingestion not reaching statistical significance (*p* = 0.051). Accordingly, the AUC of *TLR2* expression was larger after glucose ingestion when compared to those observed after lipid and protein intake (Figure 1G). These changes were not influenced by obesity or group of subjects (Figure 1H,I).

#### 3.3.2. Effect of Group and Obesity on TLR4 Expression after Macronutrient Challenges

Surface expression of TLR4 decreased significantly in monocytes and neutrophils after ingestion of all the macronutrients (Figure 2A,D and Table S2) regardless of the group of subjects (Figure 2C,F). This effect was actually related to the very large decrease observed in non-obese subjects, because the decrease observed in obese participants was much smaller (Figure 2B,E and Table S2). On the contrary, *TLR4* gene expression increased after glucose, lipid, and protein ingestion, even though the later increase did not reach statistical significance, yet no differences in the AUCs of these responses were observed (Figure 2G). Obesity and group of subjects did not influence these responses (Figure 2H,I).

#### 3.3.3. Effect of Group and Obesity on Monocyte CD36 and CD86 Expression after Macronutrient Challenges

Monocyte CD36 surface expression decreased in response to glucose and lipid intake, but not after protein ingestion (Figure 3A and Table S3). The largest AUC of CD36 surface expression was observed after the lipid challenge and the smallest after protein intake—difference that was statistically significant—with glucose ingestion inducing an intermediate response (Figure 3A). Postprandial CD36 surface expression was not globally influenced by group (Figure 3C), but we observed a larger decrease in non-obese subjects compared with their obese counterparts, particularly after lipid and glucose intake (Figure 3B).

Surface expression of CD86 in monocytes markedly decreased during the postprandial period regardless of the macronutrient ingested (Figure 3D and Table S3). The decrease showed a tendency (*p* = 0.087) to be greater after lipid than after protein ingestion (Figure 3D), a tendency (*p* = 0.094) to be larger in non-obese subjects than in obese individuals (Figure 3E) and a tendency (*p* = 0.073) to be greater in men compared with control women (Figure 3F).

**Figure 4:**
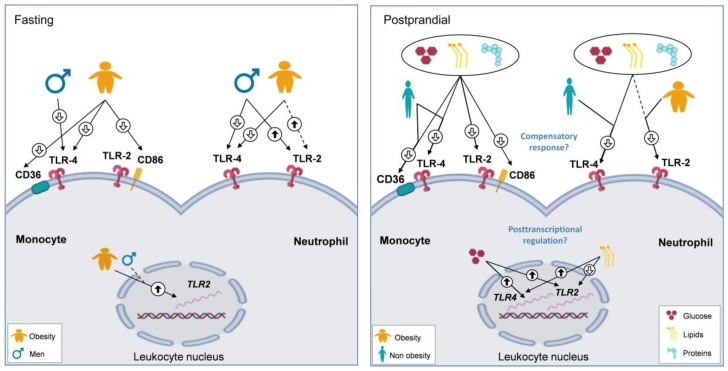
Cell-surface and gene expression of leukocyte activation markers in the fasting and postprandial states. We observed an opposite influence of obesity and sex on surface expression of TLR2 and TLR4 after fasting, and a general decrease in the surface expression after ingestion of all macronutrients which was inversely associated with their respective fasting levels. These results suggest a transient compensatory response to macronutrient intake—which is modulated by obesity—aiming to prevent an exaggerated inflammatory process. However, glucose and lipids, but not proteins, differently and effectively activated leukocyte *TLR2* and *TLR4* gene expression regardless of obesity and sex, suggesting that the intake of proteins contributes minimally to postprandial inflammation, and that obesity and sex hormones may exert their postprandial influences at the post-transcriptional level.

Finally, a summary of the findings on cell-surface and gene expression of leukocyte activation markers at the fasting and postprandial situations is presented in Figure 4.

## 4. Discussion

Our present results showed that obesity, sex, and sex hormones influenced TLR surface expression. Obese participants showed lower fasting TLR4 expression on both monocytes and neutrophils, and a tendency towards higher TLR2 expression in neutrophils, when compared with non-obese individuals. In addition, men presented higher neutrophil TLR2 expression than women, but decreased TLR4 expression compared with PCOS patients. We also observed that the postprandial phase was characterized by a decrease in the cell-surface expression of all evaluated markers, and that the type of macronutrient being administered had a smaller impact on this response. Moreover, such response was inversely correlated with the levels of surface expression observed at fasting. Those individuals who presented higher surface levels at fasting were those who showed a larger reduction postprandially. Actually, after macronutrient ingestion, neutrophil expression of TLR2 was reduced in obese subjects, and almost in men, but showed no changes in non-obese individuals and women. Conversely, postprandial surface TLR4 reduction was much more evident in non-obese participants and also in PCOS women (even though the large individual variation observed precluded that the differences between the groups of subjects reached statistical significance).

Regarding leukocyte gene expression, *TLR2* mRNA was induced after glucose ingestion but decreased in response to the oral lipid challenge. On the contrary, *TLR4* expression was greatly activated by glucose and to a lesser extent by lipids, whereas proteins caused no significant changes in either gene. However, sex, sexual steroids, and obesity did not influence *TLR2* and *TLR4* expression postprandially, despite obese subjects, chiefly men, showing higher fasting *TLR2* levels than non-obese individuals.

In accordance with the literature, our present data further support an association of TLRs and obesity [9,10,11]. In our series of young adults, obesity increased fasting TLR2 surface expression but decreased that of TLR4. This opposite pattern of expression may be related to the low-grade chronic inflammatory process associated with obesity. We may speculate that the reduced surface expression of TLR4 may represent a compensatory mechanism—similar to that observed in endotoxin tolerance [41,42]—aiming to prevent further inflammatory responses in individuals that, because of their obesity, are already susceptible to develop prionflammatory responses.

TLR2 and TLR4 are subjected to different regulatory mechanisms and are activated by different ligands. In particular, lipopolysaccharide (LPS) binds specifically to TLR4 but not TLR2 [3] and intestinal micro biota exerts a significant effect on the host immune response [11]. LPS levels have been reported to be increased in obesity as the result of increased gut permeability in response to high fat diet, and leakage of gut micro biota [43]. Persistent presence of LPS, even at low levels, may cause endotoxin tolerance, which has been associated with down regulation of cell-surface TLR4 [41,42]. Thus, a similar mechanism might account for the decreased TLR4 surface levels in our obese patients as a result of periodic hyperglycemic, hyperlipidemic, and hyper caloric insults. Such compensatory mechanism might be lost during the progression to worse inflammatory stages, which might partially explain the discrepancies observed between our present results derived from healthy young individuals and those reported in older individuals with diabetes and cardiovascular disease [44,45].

In addition to obesity, sex steroid hormones also play a key role in the modulation of bacterial-host interactions and inflammation, and have been reported to influence TLR expression [19,46]. In fact, sex divergences concerning different TLR-family members and their mediated responses have been described in humans [47,48,49]. Several studies have also described an inhibitory effect of testosterone on TLR4 expression [50,51], in accordance with men presenting with reduced TLR4 surface expression in our study. On the contrary, estrogens reduced TLR2 surface expression and promoted inflammation upon subsequent TLR4 induction [52,53], and men in our series had increased TLR2 surface expression in addition to the lowest estradiol concentrations of all groups. Thus, our data are in good agreement with previously reports highlighting the importance of sex hormones in regulating the immune-inflammatory response.

However, the literature regarding TLR expression in patients with PCOS is scarce. Two studies presented data describing increased TLR4 in ovarian tissues from animal models of PCOS [54,55], and it has been reported that ovarian cumulus cells from women with PCOS presented an abnormal TLR expression [56]. Gonzalez et al. [57,58] recently reported increased TLR2 expression in peripheral blood mononuclear cells in women with PCOS after ingestion of cream regardless of obesity, and a similar increase in TLR4 expression that occurred mostly in obese women with the syndrome. On the contrary, we have not been able to find differences among women with PCOS and control women in the TLRs expression profiles. This discrepancy may rely on the fact that the PCOS women in our series differed from controls mostly in their hyperandrogenic milieu but not in markers of adiposity and insulin resistance, characteristics strongly associated with TLR expression. Moreover, the lipid emulsion used here contained much less saturated fat (10.6% saturated fatty acids, 60.8% monounsaturated fatty acids, and 28.6% polyunsaturated fatty acids) compared with the cream used in the study by Gonzalez et al. [57,58] and saturated and polyunsaturated fatty acids have been reported to induce or protect from inflammation, respectively [59].

In general, focusing on the different macronutrient loads, surface expression of leukocyte activation markers was strongly diminished during all the macronutrient challenges with a relatively weaker decrease after protein ingestion. Upon ligand binding, TLR2 and TLR4 activation may require the internalization of the receptor leading to the down regulation of their cell-surface expression [3]. Thus, we might hypothesize that after leukocyte activation by macronutrients, during the postprandial period TLR2 and TLR4 molecules are internalized to trigger the inflammatory signaling-cascade, a mechanism that might account for the present findings. This generalized decrease might also represent a transient protective physiological response against nutrient proinflammatory stimuli, as previously hypothesized [60]. Consistently, individuals with increased fasting surface levels would further reduce their expression after macronutrient ingestion as a compensatory mechanism to counteract for possible exacerbated inflammatory responses [39]. In addition, the hyporesponsiveness observed in obese subjects regarding postprandial TLR4 expression would be in agreement with the possible endotoxin tolerance cited earlier.

We have recently reported a similar discrepancy between the generalized decrease observed in several circulating inflammatory mediators and their respective gene expression induction by macronutrients [39]. Of note, *TLR4* showed a very similar pattern of gene expression during the macronutrient challenges compared with those of interleukin 10 (*IL10*), tumor necrosis factor alpha (*TNF*), the TNF receptor superfamily member 1B (*TNFRSF1B*), and interleukin 6 receptor (*IL6R*), with the highest induction corresponding to the glucose load and to the anti-inflammatory cytokine gene *IL10* [39]. The fact that postprandial gene expression was unaffected by obesity, sex, and sex hormones suggests that any putative influence of these variables on the surface expression and circulating levels of these molecules is exerted at the posttranscriptional level. Likewise, the lack of correlation between postprandial changes in TLR mRNA and cell-surface expression suggests that TLR-mediated signaling responses are highly regulated pathways. Indeed, it has been described that IL10 may exert its action by inhibiting the translocation of surface receptors from their intracellular reservoirs to the cell-membrane [61], and although cell-surface expression of TLRs was decreased after macronutrient loads, activation of TLRs may also account intracellularly [62]. Furthermore, absence of changes in gene expression must not be taken as evidence of absence of physiologically relevant changes [63,64].

The impact of macronutrients on leukocyte function and its association with inflammation have been reported earlier, but most of the experiments addressing their effects on TLR expression have been performed either in vitro [27] or following the ingestion of mixed meals [65,66,67]. Even with some discrepancies, most of these studies showed that glucose and fat ingestion increased the expression of TLR2 and TLR4 [65,66,67]. However, data regarding the effects of protein ingestion are emerging [28,30,31] and, to the best of our knowledge, only one study has evaluated the impact of dietary protein in women with PCOS [29]. Although our results demonstrated the same generalized profile of reduced surface expression of leukocyte markers during the postprandial phase, the oral protein load seemed to cause a lesser decrease and, interestingly, its effect on gene expression was much weaker, or in some cases inexistent, confirming the lesser influence of this macronutrient on leukocyte activation compared with glucose and lipids. In our experiments, the glucose load was by far the challenge that induced a greater gene expression for both *TLR2* and *TLR4*, in agreement with several in vitro studies [65,68,69]. Moreover, it has been reported that TLR overexpression in hyper-glycaemic conditions is actually linked to increased oxidative stress [27]. We recently reported that only the oral glucose challenge caused an elevation in plasma TBARS concentrations (an index of lipid peroxidation and oxidative damage), and that obese men presented also with increased TBARS levels during fasting conditions [34]. This data would be in accordance with our present results showing that only obese men showed higher fasting *TLR2* mRNA expression compared with their non-obese counterparts. Finally, the lack of *TLR2* induction by lipids in our study might be related to the large percentage of mono- and polyunsaturated fatty acids in the lipid emulsion administered to our experimental subjects, as exposed earlier and consistent with previous reports demonstrating an induction of TLR expression and proinflammatory cytokine release by palmitate and stereate but not with oleate or palmitoleate [70,71].

Our results concerning monocyte CD36 and CD86 expression suggest an influence of obesity. Remarkably, CD36 and CD86 surface expression showed strong correlations with those of TLR4 and TLR2, respectively. Both TLRs have been associated with CD36 in several studies [4,5] and postulated to induce CD86 expression [6,7]. CD36 has also been reported to decrease in monocytes following exposure to LPS in an endotoxin tolerance-like manner as suggested for TLR4 [42]. There is some evidence that hyperglycemic conditions induce transient changes in monocyte CD36 surface expression [72]. Of note, after protein ingestion CD36 levels showed no reduction while CD86 strongly decreased, particularly in men. Therefore, the regulatory mechanisms influencing their postprandial expression may be differently modulated, at least for proteins, as has been recently described for other surface markers [32]. This latter study did not report significant changes of *CD36* and *CD86* gene expression after macronutrient intake, nevertheless such work only included younger and lean men and its lipid and protein loads differed from ours in macronutrient source and composition [32]. As for TLR, we hypothesized that the decreased surface expression following macronutrient ingestion may account for a physiologic compensatory mechanism aiming to avoid an exaggerated inflammatory response.

However, our study was subjected to certain limitations. We have not performed complementary and in vitro analyses that would have provided valuable information and greater robustness to our results. Due to technical reasons, fluorescent markers for TLRs and CD proteins could not be combined in the flow cytometry experiments. Gene expression assays were carried out in whole white blood cells, therefore, we could not conduct direct correlations with surface levels nor differentiate the true expression in each different leukocyte subpopulation, which hinder a clearer interpretation of the results. Moreover, given the complexity of our study design, the sample size of the subgroups was relatively small because of the need to perform three independent oral loads in alternate days and, thus, our study may have been underpowered to detect small differences among groups of subjects. Finally, the order of macronutrient challenges was not randomized, yet the possibility of carryover effects was minimized by conducting the oral loads in alternate days. In our opinion, such limitations were compensated by the rather homogeneous healthy population studied here in terms of age and percentage of obesity, the recommendation to follow the same diet for a few days before the start of the study, the quality of the procedures used in the challenges, and the administration of exactly the same number of calories in the three oral loads.

## 5. Conclusions

We observe a general decrease in the cell-surface expression of leukocyte activation markers after ingestion of either macronutrient that is inversely associated with their expression at fasting, and an opposite influence of obesity and sex on the cell-surface expression of TLR2 and TLR4 in leukocytes. These results suggest a transient compensatory response of immune cells to macronutrient intake aiming to prevent an exacerbated inflammatory process, which is modulated by obesity. However, glucose and lipids, but not proteins, differently and effectively activated leukocyte *TLR2* and *TLR4* gene expression regardless of obesity, sex, or sex hormones, suggesting a lesser effect of protein intake on postprandial inflammation, and that the mechanisms regulating this compensatory response appear to occur at the posttranscriptional level. Additional investigations are required to further discern the specific mechanisms by which macronutrients, obesity, and sex hormones exert their different effects on the immune cell response and the role of TLR-regulation in these processes.

## Figures and Tables

**Table 1:** Cell-surface expression of TLR-2, TLR-4, CD36 and CD86 on monocytes and neutrophils, and gene expression of *TLR2* and *TLR4* in leukocytes, after fasting.

	Control Women						PCOS Women						Control Men						Group	Obesity	Interaction
	Non-Obese			Obese			Non-Obese			Obese			Non-Obese			Obese			*p* Value	*p* Value	*p* Value
***Monocyte Expression***	(n = 6)			(n = 7)			(n = 7)			(n = 5)			(n = 10)			(n = 9)					
**TLR-2**	15.2	±	7.2	13.0	±	1.8	13.2	±	4.2	13.8	±	5.9	17.7	±	9.5	14.4	±	2.9	0.365	0.545	0.810
**TLR-4 ^b^**	14.1	±	10.4	7.4	±	11.1	25.7	±	28.0	6.8	±	4.3	8.9	±	9.7	3.3	±	1.0	0.042	0.001	0.583
**CD36**	42.8	±	34.6	17.4	±	30.5	76.4	±	74.1	13.7	±	8.0	54.1	±	73.1	5.8	±	4.0	0.122	<0.001	0.990
**CD86**	5.3	±	1.4	4.2	±	0.5	5.2	±	2.1	4.0	±	0.7	5.4	±	1.6	4.3	±	0.8	0.908	0.011	0.998
***Neutrophil Expression***	(n = 6)			(n = 7)			(n = 7)			(n = 5)			(n = 10)			(n = 9)					
**TLR-2 ^a,b^**	2.3	±	0.7	2.9	±	0.7	2.3	±	0.6	2.7	±	0.8	3.1	±	0.7	3.3	±	0.7	0.015	0.077	0.690
**TLR-4 ^b^**	6.1	±	4.6	4.0	±	3.9	9.3	±	7.8	3.6	±	2.1	4.9	±	5.0	1.9	±	0.3	0.043	0.002	0.890
***Leukocyte Gene Expression***	(n = 9)			(n = 8)			(n = 9)			(n = 8)			(n = 10)			(n = 9)					
***TLR2***	0.08	±	0.08	0.05	±	0.10	0.00	±	0.11	0.08	±	0.16	−0.05	±	0.14	0.12	±	0.12	0.774	0.034	0.072
***TLR4***	−0.48	±	0.15	−0.54	±	0.16	−0.53	±	0.10	−0.39	±	0.12	−0.55	±	0.21	−0.45	±	0.15	0.642	0.162	0.168

Data are means ± SD and represent the mean value of fasting levels prior to each macronutrient load. Data are expressed as arbitrary units (MFI ratio for flow cytometry and log2^−ΔCq^ for gene expression). The following specific fluorescent markers were used to identify monocytes (CD14-FITC, CD33-PE and CD45-PerCP) and neutrophils (CD33-PE and CD45-PerCP) by flow cytometry. Data were analyzed by univariate general linear models and the least significant difference post-hoc test. ^a^ *p* < 0.05 for the difference between men and control women, independently of obesity. ^b^ *p* < 0.05 for the difference between men and PCOS women, independently of obesity.

**Table 2:** Correlations between fasting cell-surface expression or gene expression and their respective AUC after macronutrient challenges.

	Monocyte Surface Expression								Neutrophil Surface Expression				Leukocyte Gene Expression			
	TLR-2		TLR-4		CD36		CD86		TLR-2		TLR-4		*TLR2*		*TLR4*	
	(n = 35)		(n = 37)		(n = 35)		(n = 35)		(n = 36)		(n = 37)		(n = 53)		(n = 53)	
**Oral Load**	r	*p*	*ρ*	*p*	*ρ*	*p*	r	*p*	r	*p*	*ρ*	*p*	r	*p*	r	*p*
**Glucose**	−0.845	<0.001	−0.741	<0.001	−0.514	0.002	−0.765	<0.001	−0.479	0.002	−0.861	<0.001	−0.397	0.003	−0.387	0.004
**Lipids**	−0.847	<0.001	−0.775	<0.001	−0.532	0.001	−0.820	<0.001	−0.656	<0.001	−0.915	<0.001	−0.539	<0.001	−0.540	<0.001
**Proteins**	−0.620	<0.001	−0.911	<0.001	−0.592	0.001	−0.721	<0.001	−0.221	0.101	−0.921	<0.001	−0.212	0.128	−0.339	0.013

Data were submitted to Pearson’s (r) or Spearman’s (*ρ*) correlation analysis as appropriate.

## Supplementary Materials

The following are available online at https://www.mdpi.com/2218-273X/10/1/111/s1. Supplementary Methods: Oral macronutrient challenges, leukocyte isolation, quality control, and relative quantification; Figure S1: Gating strategy for leukocyte populations and TLR2 surface expression from one representative participant; Table S1: Clinical, biochemical, and hormonal characteristics of participants; Table S2: Cell-surface expression of TLR2 and TLR4 on monocytes and neutrophils at baseline and after the glucose, lipid, and protein oral loads; Table S3: Cell-surface expression of CD36 and CD86 on monocytes at baseline and after the glucose, lipid, and protein oral loads; Table S4: Correlations among cell-surface expression of TLR2, TLR4, CD36, and CD86 after fasting and with clinical and biochemical variables, considering all subjects as a whole.

## Abbreviations

AUC: Area under the curve; BMI: Body mass index; CD: Cluster of differentiation; GLM: General linear model; HDL: High density-lipoprotein; HOMA-IR: Homeostasis model assessment of insulin resistance; hsCRP: High-sensitivity C-reactive protein; ISI: Insulin sensitivity index; LDL: Low density-lipoprotein; MFI: Mean fluorescence intensity; PCOS: Polycystic ovary syndrome; qPCR: Quantitative real-time polymerase chain reaction; SHBG: Sex hormone-binding globulin; TLR: Toll-like receptors.
