# Supplementary material for: TLR2 and TLR4 Surface and Gene Expression in White Blood Cells after Fasting and Oral Glucose, Lipid and Protein Challenges: Influence of Obesity and Sex Hormones
Source: Biomolecules. 2020 Jan 9;10(1):111. doi: 10.3390/biom10010111 (PMC7023426; doi:10.3390/biom10010111)
Supplement: Supplementary file 1 [file biomolecules-10-00111-s001.pdf]

SUPPLEMENTARY METHODS

Oral macronutrient challenges

On alternate days, we submitted patients to oral loads of glucose, lipids and protein. The quantities and volumes in the oral loads were adjusted for a total caloric intake of 300 kcal each, irrespective of the macronutrient being administered. Therefore, we administered 200 ml of a 37.5 g/dl glucose solution (GlycoSull Naranja 75 g, Química Clínica Aplicada, Spain) for the oral glucose load, 66 ml of a 4.5 kcal/ml long-chain triglyceride enteral nutrition supplement (Supracal neutro, Nutricia S.R.L., Spain) for the oral lipid load, and 75 g of an enteral nutrition supplement containing caseinates (Proteína NM, Nutrición Médica S.L., Spain) for the oral protein load. The composition of the lipid supplement was 10.6%, 60.8% and 28.6% of saturated, mono, and poly unsaturated fatty acids, respectively. Whereas we observed no problem with the ingestion of the glucose and lipid supplements, the high viscosity and relatively poor palatability of the protein supplement made difficult the administration and ingestion of the same amounts of kcal in all the subjects. Hence, we calculated the amounts of kcal ingested in 21 consecutive protein preparations, after discounting from the initial amount the quantities lost in the preparation and the remnant that was not ingested, resulting into a mean of 304.7 kcal ingested per subject with a 0.05 coefficient of variation.

Leukocyte isolation

Peripheral blood leukocytes were immediately obtained after blood draw from EDTA anticoagulated blood samples after incubation for 60 minutes with cold dextran 3% (1:1) at 4ºC. Cells in the supernatant were diluted in NaCl 0.9% up to 50 ml and centrifuged at 525 x g for 10 min at 4ºC. Cells were then lysed by osmotic shock during 50 seconds and subsequently washed twice at 325xg and 250xg in NaCl 0.9% at 4ºC. Cell pellets were resuspended in cold PBS, counted in a Neubauer chamber and finally centrifuged at 250xg for 10 min to be stored in RNAlater (Sigma-Aldrich Química S.A., Alcobendas, Spain) at -80ºC.

Quality control and relative quantification (qPCR)

RNA yield, quality and purity were assessed with a NanoDrop2000 spectrophotometer and the Qubit RNA HS Assay kit in a Qubit3.0 fluorimeter (ThermoFisher Scientific – Spain). We randomly selected more than 30 samples to evaluate the RNA integrity number (RIN) with the High Sensitivity RNA ScreenTape in a TapeStation2200 (Agilent Technologies). All the samples evaluated presented RINs > 8.5.

To test our samples for factors that could lead to unreliable results in SYBR Green qPCR we evaluated 24 unselected samples with the Human RT2 RNA QC PCR array (Qiagen, Hilden, Germany) prior to proceeding with complete gene expression experiments. We also evaluated seven genes (*B2M*, *GUSB*, *ACTB*, *HPRT1*, *TBP*, *RPS18* and *GADPH*) to determine their usability as reference genes. *RPS18* and *HPRT1* showed a more consistent expression in our samples and therefore were selected as reference genes for data normalization (Primer sets, buffer and enzyme are available from AnyGenes, Paris, France). For the relative quantification of gene expression levels we used the comparative threshold cycle method [73]. A quantification cycle (Cq) was obtained for each amplification curve and a ΔCq value was first calculated by subtracting the average Cq value of *RPS18* and *HPRT1* from the Cq value of the gene of interest. Data were expressed as arbitrary units using the following transformation [expression = $\log 2^{-\Delta Cq}$].

73. Livak, K.J.; Schmittgen, T.D. Analysis of relative gene expression data using real-time quantitative PCR and the 2(-Delta Delta C(T)) Method. *Methods* **2001**, *25*, 402-408.

Figure S1. Gating strategy for leukocyte populations and TLR-2 surface expression from one representative participant. (**A**) Leukocytes were defined according to the forward-side scatter plot in human whole blood. (**B**) Monocytes and neutrophils were identified and gated according to their characteristic side-scatter flow cytometry profile and their expression of CD45+, excluding cellular debris. A combination of CD14 or CD33 with TLR2/TLR4 or CD36/CD86 was also used to improve monocyte and neutrophil identification. (**C** & **D**) Using the isotype control sample, gates were set so that at least 98% of isotype control staining were negative for each specific marker expression, both in the monocyte and neutrophil populations. Cut-off quadrant markers based on the isotype control were set individually for each measurement. (**E** & **F**) Overlay fluorescence histograms showing the expression of isotype-control-FITC (grey histogram) and TLR2-FITC (black histogram) on monocytes and neutrophils.

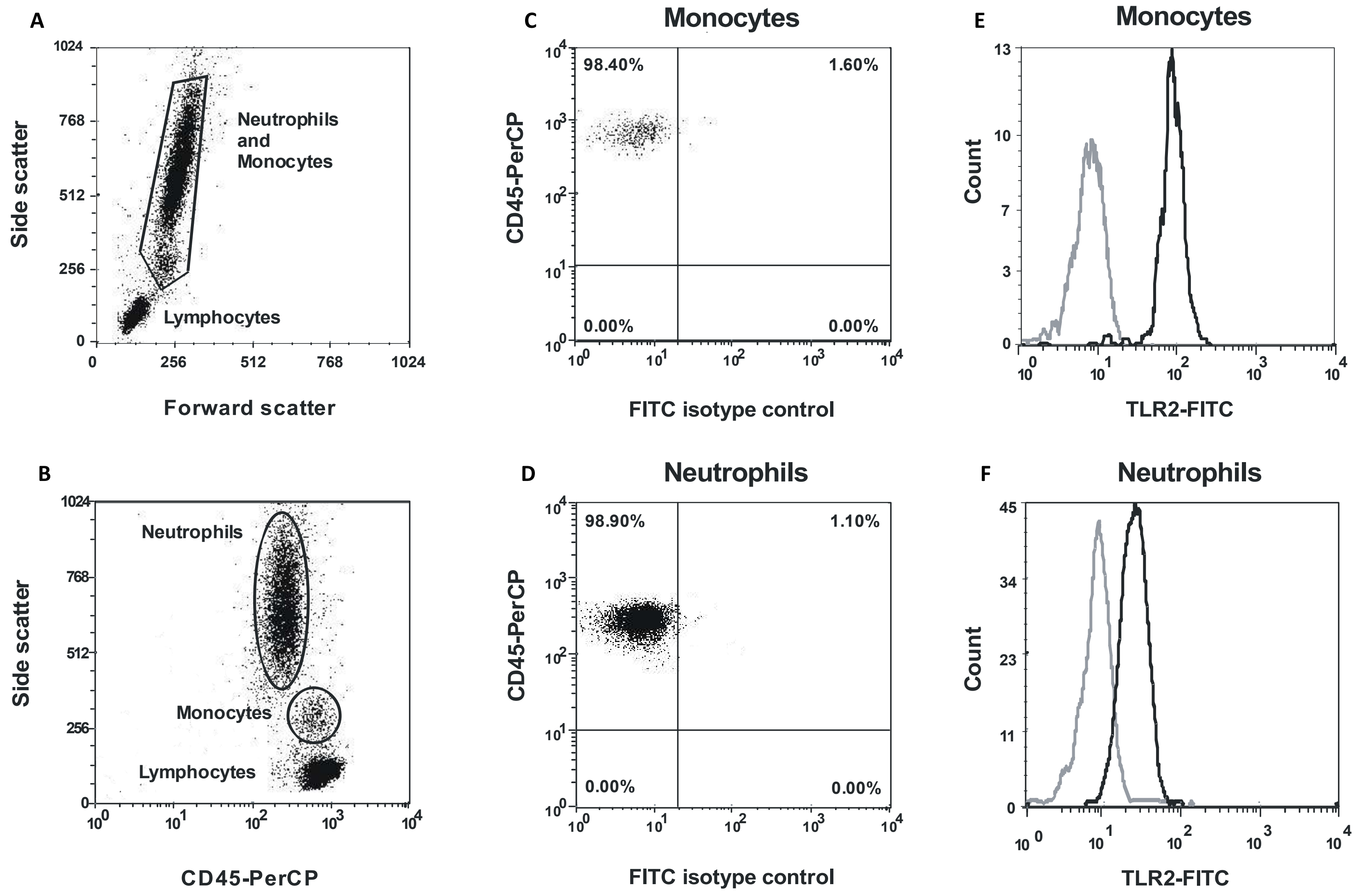

Table S1. Clinical, biochemical, and hormonal characteristics of participants.

Variable	Control women (n = 17)		PCOS women (n = 17)		Control men (n = 19)		Group	Obesity	Interaction
	Non-obese (n = 9)	Obese (n = 8)	Non-obese (n = 9)	Obese (n = 8)	Non-obese (n = 10)	Obese (n = 9)	*P* value	*P* value	*P* value
Age (yr-old)	26 ± 5	27 ± 6	24 ± 8	30 ± 5	24 ± 4	25 ± 4	0.342	0.101	0.319
Body mass index (kg/m^2)	23 ± 2	36 ± 4	24 ± 3	37 ± 5	23 ± 2	34 ± 3	0.226	<0.001	0.782
Waist circumference (cm)[a,b]	76 ± 9	100 ± 17	72 ± 7	105 ± 11	81 ± 5	110 ± 13	0.050	<0.001	0.377
Waist-to-hip ratio [a,b]	0.75 ± 0.08	0.83 ± 0.12	0.73 ± 0.05	0.85 ± 0.06	0.83 ± 0.04	0.90 ± 0.05	0.002	<0.001	0.436
Hirsutism score	1.4 ± 1.3	1.8 ± 1.2	9.7 ± 4.5	9.3 ± 4.5	-	-	<0.001	0.738	0.876
Systolic blood pressure (mm Hg)	117 ± 10	122 ± 15	111 ± 15	121 ± 16	123 ± 13	128 ± 12	0.122	0.087	0.845
Diastolic blood pressure (mm Hg)	75 ± 10	81 ± 10	72 ± 10	81 ± 9	69 ± 7	77 ± 6	0.196	0.002	0.800
Total T (nmol/l) [a,b,c]	1.6 ± 0.3	2.0 ± 0.5	2.5 ± 0.7	2.4 ± 1.0	18.5 ± 3.3	17.3 ± 3.6	<0.001	0.776	0.196
Total E_2 (pmol/l) [a,b]	149 ± 63	276 ± 200	182 ± 201	149 ± 49	68 ± 16	94 ± 26	<0.001	0.024	0.422
Free T (pmol/l) [a,b,c]	21 ± 7	31 ± 8	36 ± 12	45 ± 24	450 ± 104	464 ± 94	<0.001	0.024	0.265
Free E_2 (pmol/l) [a]	2.7 ± 1.1	5.3 ± 3	3.6 ± 4.3	3.4 ± 1.4	1.8 ± 0.5	2.6 ± 0.7	0.010	0.003	0.520
Ratio Free T/Free E_2 [a,b,c]	8.6 ± 0.9	7.6 ± 1.6	17.4 ± 4.0	14.0 ± 1.9	263.4 ± 22.6	186.4 ± 12.7	<0.001	0.119	0.750
SHBG (nmol/l) [a,b]	56 ± 25	43 ± 14	50 ± 21	32 ± 13	27 ± 10	20 ± 6	<0.001	0.008	0.568
Androstenedione (nmol/l) [b,c]	9.1 ± 2.9	9.5 ± 2.9	14.7 ± 4.2	13.4 ± 6.1	7.1 ± 1.7	9.3 ± 3.8	<0.001	0.712	0.371
DHEAS (µmol/l)	5.4 ± 1.2	5.3 ± 1.5	7.6 ± 3.9	5.7 ± 2.1	6.0 ± 1.8	8.2 ± 2.9	0.094	0.852	0.081
Ferritin (pmol/l) [a,b]	75 ± 73	90 ± 50	90 ± 67	107 ± 53	260 ± 267	441 ± 397	<0.001	0.047	0.936
WBC count (x10^9/l)	5284 ± 1435	6360 ± 1326	5744 ± 2587	7768 ± 1974	4973 ± 1109	6353 ± 1186	0.126	0.002	0.704
Neutrophil count (x10^9/l)	3127 ± 1098	3773 ± 858	3329 ± 2175	4631 ± 1349	2592 ± 711	3682 ± 839	0.146	0.006	0.745
Monocyte count (x10^9/l)	436 ± 123	484 ± 143	460 ± 296	511 ± 160	418 ± 122	413 ± 105	0.466	0.505	0.857
Lymphocyte count (x10^9/l)	1604 ± 372	1931 ± 410	1782 ± 341	2360 ± 606	1759 ± 400	2008 ± 545	0.154	0.003	0.535
hsCRP (nmol/l)	27 ± 22	38 ± 28	20 ± 21	65 ± 74	31 ± 25	31 ± 12	0.998	0.009	0.207
Triglycerides (mmol/l)	0.84 ± 0.36	0.92 ± 0.34	0.90 ± 0.25	1.14 ± 0.47	0.89 ± 0.29	1.15 ± 0.40	0.399	0.054	0.711
Total cholesterol (mmol/l)	4.4 ± 0.9	4.7 ± 0.9	4.3 ± 1.0	4.4 ± 0.9	4.1 ± 0.6	4.8 ± 0.9	0.844	0.133	0.485
HDL-cholesterol (mmol/l) [a,b]	1.4 ± 0.3	1.3 ± 0.2	1.4 ± 0.2	1.2 ± 0.2	1.2 ± 0.2	1.0 ± 0.1	0.001	0.002	0.512
LDL-cholesterol (mmol/l)	2.7 ± 0.8	2.9 ± 0.6	2.5 ± 0.8	2.7 ± 0.8	2.4 ± 0.4	3.3 ± 0.8	0.507	0.034	0.237
Fasting insulin (pmol/l)	55 ± 20	77 ± 21	52 ± 29	94 ± 25	40 ± 11	75 ± 27	0.123	<0.001	0.440
Fasting glucose (mmol/l) [b,c]	4.7 ± 0.4	5.3 ± 0.4	4.5 ± 0.5	4.8 ± 0.5	4.9 ± 0.5	5.2 ± 0.4	0.011	0.001	0.408
Insulin sensitivity index	6.6 ± 2.8	3.3 ± 1.2	8.1 ± 4.7	3.6 ± 1.4	7.3 ± 2.8	3.8 ± 1.6	0.640	<0.001	0.909
HOMA-IR	1.6 ± 0.6	2.6 ± 0.7	1.5 ± 0.9	2.9 ± 0.7	1.3 ± 0.4	2.5 ± 1.0	0.422	<0.001	0.744

Abbreviations: DHEAS, dehydroepiandrosterone sulfate; E_2, estradiol; hsCRP, high-sensitivity C-reactive protein; HOMA-IR, homeostasis model assessment of insulin resistance; SHBG, sex hormone-binding globulin; T, testosterone; WBC, white blood cells.

Data are means ± SD. Data were analyzed by univariate general linear models followed by the least significant difference post-hoc test. [a]*P* < 0.05 for the difference between men and control women, independently of obesity. [b]*P* < 0.05 for the difference between men and PCOS women, independently of obesity. [c]*P* < 0.05 for the difference between PCOS and control women, independently of obesity.

Table S2. Cell-surface expression of TLR-2 and TLR-4 on monocytes and neutrophils at baseline and after the glucose, lipid and protein oral loads.

		Control women		PCOS women		Control men		*P* value	*P* value	*P* value
		Non-obese (n = 6)	Obese (n = 6)	Non-obese (n = 6)	Obese (n = 5)	Non-obese (n = 7)	Obese (n = 7)	Oral load	Oral load x Group	Oral load x Obesity
Monocyte expression										
	Glucose Load (0 h)	12.8 ± 4.0	13.1 ± 2.7	12.7 ± 3.3	13.9 ± 6.5	10.3 ± 13.0	13.1 ± 2.7			
	Glucose Load (2 h)	10.2 ± 1.9	10.9 ± 2.5	11.0 ± 2.3	11.6 ± 4.6	14.8 ± 8.4	9.7 ± 1.5	<0.001	0.204	0.968
	Glucose Load (2 h-0 h)	-2.7 ± 3.7	-2.1 ± 2.7	-1.7 ± 2.9	-2.3 ± 2.2	-5.4 ± 4.8	-3.4 ± 1.8			
	Lipid Load (0 h)	17.0 ± 6.8	13.3 ± 1.7	12.8 ± 5.4	17.2 ± 5.9	20.3 ± 11.6	15.3 ± 3.2			
**TLR-2**	Lipid Load (4 h)	12.8 ± 4.7	11.6 ± 1.5	10.7 ± 3.5	12.5 ± 2.8	14.2 ± 5.8	10.2 ± 1.1	<0.001	0.042	0.512
	Lipid Load (4 h-0 h)	-4.2 ± 4.1	-1.7 ± 1.1	-2.1 ± 2.1	-4.7 ± 3.5	-6.1 ± 6.1	-5.2 ± 2.3			
	Protein Load (0 h)	15.9 ± 11.9	12.8 ± 2.6	13.4 ± 6.4	14.9 ± 4.6	17.9 ± 9.6	13.6 ± 3.2			
	Protein Load (2 h)	14.5 ± 12.7	10.5 ± 2.0	11.3 ± 4.0	11.3 ± 2.2	13.6 ± 6.7	9.7 ± 2.8	<0.001	0.173	0.113
	Protein Load (2 h-0 h)	-1.4 ± 1.5	-2.4 ± 2.2	-2.1 ± 3.1	-3.6 ± 2.6	-4.3 ± 3.8	-3.9 ± 1.7			
	Glucose Load (0 h)	16.6 ± 13.5	4.9 ± 7.5	28.7 ± 29.1	6.7 ± 5.3	10.4 ± 9.9	3.7 ± 1.1			
	Glucose Load (2 h)	9.2 ± 2.8	6.0 ± 9.7	17.4 ± 15.9	5.6 ± 3.2	7.8 ± 8.1	3.2 ± 0.9	<0.001	0.693	0.001
	Glucose Load (2 h-0 h)	-7.3 ± 10.8	1.0 ± 2.2	-11.3 ± 14.2	-1.1 ± 2.2	-2.5 ± 2.0	-0.4 ± 0.8			
	Lipid Load (0 h)	14.2 ± 9.3	5.9 ± 9.5	31.1 ± 34.2	7.4 ± 5.4	10.3 ± 10.1	3.5 ± 0.9			
**TLR-4**	Lipid Load (4 h)	10.3 ± 4.3	5.4 ± 8.4	16.6 ± 16.1	5.5 ± 3.6	7.8 ± 7.6	2.7 ± 0.6	<0.001	0.122	0.105
	Lipid Load (4 h-0 h)	-3.8 ± 5.3	-0.5 ± 1.2	-14.5 ± 21.5	-2.0 ± 2.2	-2.5 ± 2.6	-0.7 ± 0.7			
	Protein Load (0 h)	11.6 ± 9.5	9.6 ± 18.9	25.6 ± 26.8	5.7 ± 4.3	10.2 ± 11.5	3.3 ± 0.9			
	Protein Load (2 h)	9.9 ± 8.5	6.4 ± 10.6	15.6 ± 15.2	4.6 ± 3.0	9.0 ± 9.4	2.7 ± 0.7	0.008	0.225	0.668
	Protein Load (2 h-0 h)	-1.8 ± 1.8	-3.2 ± 8.3	-9.9 ± 12.3	-1.1 ± 1.4	-1.2 ± 2.5	-0.5 ± 0.5			
Neutrophil expression										
	Glucose Load (0 h)	2.2 ± 0.7	3.0 ± 0.4	2.1 ± 0.3	2.4 ± 0.9	3.2 ± 0.9	3.4 ± 0.6			
	Glucose Load (2 h)	2.4 ± 0.6	2.6 ± 0.6	2.2 ± 0.4	2.5 ± 0.8	3.1 ± 1.2	2.5 ± 0.7	0.149	0.052	0.028
	Glucose Load (2 h-0 h)	0.2 ± 0.9	-0.4 ± 0.7	0.2 ± 0.4	0.1 ± 0.3	-0.1 ± 0.6	-0.9 ± 0.5			
	Lipid Load (0 h)	2.4 ± 1.0	3.2 ± 1.0	1.9 ± 0.4	3.0 ± 0.8	3.4 ± 1.0	3.5 ± 1.0			
**TLR-2**	Lipid Load (4 h)	2.5 ± 0.9	2.8 ± 0.7	2.4 ± 0.7	2.9 ± 0.5	3.3 ± 1.2	2.6 ± 0.5	0.308	0.137	0.043
	Lipid Load (4 h-0 h)	0.1 ± 0.9	-0.4 ± 1.0	0.4 ± 0.4	-0.0 ± 0.6	-0.1 ± 1.2	-1.0 ± 0.6			
	Protein Load (0 h)	2.6 ± 1.5	2.9 ± 0.7	2.5 ± 1.1	2.8 ± 0.9	3.2 ± 0.9	3.1 ± 0.8			
	Protein Load (2 h)	2.8 ± 2.0	2.3 ± 0.3	2.8 ± 1.3	2.7 ± 0.2	3.0 ± 1.2	2.5 ± 0.8	0.092	0.217	0.022
	Protein Load (2 h-0 h)	0.2 ± 0.6	-0.6 ± 0.7	0.3 ± 0.3	-0.2 ± 0.9	-0.2 ± 0.8	-0.6 ± 0.3			
	Glucose Load (0 h)	6.7 ± 5.7	4.3 ± 4.3	10.0 ± 8.8	3.4 ± 2.4	5.9 ± 5.2	2.0 ± 0.4			
	Glucose Load (2 h)	3.6 ± 1.7	2.9 ± 3.5	5.6 ± 3.2	2.8 ± 1.2	3.9 ± 2.5	1.7 ± 0.3	<0.001	0.447	0.136
	Glucose Load (2 h-0 h)	-3.0 ± 4.3	-1.3 ± 2.9	-4.4 ± 6.3	-0.6 ± 1.3	-2.0 ± 3.3	-0.2 ± 0.5			
	Lipid Load (0 h)	6.9 ± 5.7	3.0 ± 3.2	10.6 ± 9.2	4.2 ± 2.5	7.1 ± 7.4	1.9 ± 0.3			
**TLR-4**	Lipid Load (4 h)	3.7 ± 1.5	2.4 ± 2.3	5.6 ± 3.3	3.3 ± 1.5	4.0 ± 3.2	1.7 ± 0.4	<0.001	0.916	0.003
	Lipid Load (4 h-0 h)	-3.2 ± 4.3	-0.5 ± 0.9	-5.0 ± 6.0	-0.8 ± 1.1	-3.1 ± 4.8	-0.2 ± 0.2			
	Protein Load (0 h)	4.7 ± 2.5	3.9 ± 5.9	9.0 ± 7.7	3.4 ± 1.7	5.4 ± 4.6	1.9 ± 0.6			
	Protein Load (2 h)	3.5 ± 1.6	2.7 ± 2.7	5.5 ± 3.2	2.9 ± 1.2	4.0 ± 3.1	1.7 ± 0.3	<0.001	0.584	0.006
	Protein Load (2 h-0 h)	-1.2 ± 1.3	-1.3 ± 3.2	-3.5 ± 4.7	-0.5 ± 0.6	-1.4 ± 1.8	-0.1 ± 0.3			

Data are means ± SD and represent the MFI ratio (MFI of the mAb of interest to the MFI of the matched isotypic negative control). Data were analyzed by univariate general linear models for repeated measures introducing fasting and postprandial data as within-subjects factor (to evaluate differences from fasting levels: *Oral load P* value), and Obesity and Group as between-subjects factor (to evaluate their influence in the postprandial response: *Oral load x Group* and *Oral load x Obesity P* values).

Table S3. Cell-surface expression of CD36 and CD86 on monocytes at baseline and after the glucose, lipid and protein oral loads.

		Control women		PCOS women		Control men		*P* value	*P* value	*P* value
		Non-obese (n = 6)	Obese (n = 6)	Non-obese (n = 6)	Obese (n = 5)	Non-obese (n = 7)	Obese (n = 7)	*Oral load*	*Oral load x Group*	*Oral load x Obesity*
Monocyte expression										
**CD36**	Glucose Load (0 h)	39.8 ± 35.1	14.1 ± 25.5	91.2 ± 78.4	14.6 ± 10.0	53.9 ± 81.8	7.6 ± 4.8			
	Glucose Load (2 h)	30.5 ± 20.9	14.0 ± 25.2	80.9 ± 66.2	12.5 ± 10.5	36.0 ± 57.8	4.8 ± 2.0	0.002	0.033	0.370
	Glucose Load (2 h-0 h)	-9.2 ± 14.6	-0.1 ± 0.9	-10.3 ± 15.3	-2.1 ± 4.9	-17.9 ± 24.3	-2.5 ± 4.0			
	Lipid Load (0 h)	55.7 ± 64.9	18.1 ± 35.7	104.9 ± 107.2	12.4 ± 8.5	49.4 ± 70.9	6.2 ± 3.4			
	Lipid Load (4 h)	35.9 ± 37.2	18.1 ± 36.9	55.6 ± 37.8	9.7 ± 5.9	32.6 ± 42.6	5.1 ± 3.1	<0.001	0.874	0.250
	Lipid Load (4 h-0 h)	-19.8 ± 27.6	-0.0 ± 1.3	-49.3 ± 72.9	-2.7 ± 3.5	-16.8 ± 28.5	-1.0 ± 1.8			
	Protein Load (0 h)	28.1 ± 32.9	19.6 ± 38.9	74.6 ± 65.4	9.2 ± 6.1	51.3 ± 75.4	5.2 ± 4.2			
	Protein Load (2 h)	22.7 ± 28.6	18.0 ± 23.5	63.7 ± 47.6	7.5 ± 3.5	52.5 ± 78.2	4.7 ± 3.3	0.773	0.675	0.221
	Protein Load (2 h-0 h)	-5.3 ± 4.9	-1.6 ± 18.3	-10.9 ± 58.2	-1.7 ± 2.7	1.2 ± 15.3	-0.4 ± 1.3			
**CD86**	Glucose Load (0 h)	5.0 ± 1.7	4.2 ± 0.17	5.6 ± 2.3	4.2 ± 0.9	6.1 ± 1.5	4.3 ± 0.9			
	Glucose Load (2 h)	3.7 ± 0.6	3.3 ± 0.7	4.3 ± 1.6	3.5 ± 0.7	4.3 ± 0.9	2.9 ± 0.5	<0.001	0.332	0.189
	Glucose Load (2 h-0 h)	-1.3 ± 1.3	-0.8 ± 0.6	-1.2 ± 1.2	-0.7 ± 0.7	-1.7 ± 1.2	-1.3 ± 0.7			
	Lipid Load (0 h)	5.9 ± 1.8	4.3 ± 0.8	5.3 ± 2.6	4.6 ± 0.7	6.3 ± 1.9	4.6 ± 0.8			
	Lipid Load (4 h)	4.2 ± 0.9	3.7 ± 0.6	3.8 ± 1.7	3.5 ± 0.2	4.2 ± 1.2	3.1 ± 0.5	<0.001	0.233	0.037
	Lipid Load (4 h-0 h)	-1.7 ± 1.3	-0.6 ± 0.6	-1.6 ± 1.3	-1.1 ± 0.6	-2.1 ± 1.0	-1.5 ± 0.8			
	Protein Load (0 h)	4.7 ± 1.4	3.8 ± 0.6	4.7 ± 2.5	3.9 ± 0.5	5.4 ± 1.5	4.4 ± 0.8			
	Protein Load (2 h)	3.9 ± 1.2	3.4 ± 0.7	3.6 ± 1.6	2.9 ± 0.2	3.9 ± 1.0	2.9 ± 0.5	<0.001	0.010	0.671
	Protein Load (2 h-0 h)	-0.7 ± 0.5	-0.5 ± 0.4	-1.1 ± 1.0	-1.0 ± 0.4	-1.5 ± 0.8	-1.5 ± 0.5			

Data are means ± SD and represent the MFI ratio (MFI of the mAb of interest to the MFI of the matched isotypic negative control). Data were analyzed by univariate general linear models for repeated measures introducing fasting and postprandial levels as within-subjects factor (to evaluate differences from fasting levels: *Oral load P* value), and Obesity and Group as between-subjects factor (to evaluate their influence in the postprandial response: *Oral load x* Group and *Oral load x Obesity P* values).

Table S4. Correlations among cell-surface expression of TLR-2, TLR-4, CD36 and CD86 at fasting and with clinical and biochemical variables, considering all subjects as a whole (n = 44).

		Surface expression in monocytes								Surface expression in neutrophils			
		**TLR-2**		**TLR-4**		**CD36**		**CD86**		**TLR-2**		**TLR-4**	
		r	(*p*)	r	(*p*)	r	(*p*)	r	(*p*)	r	(*p*)	r	(*p*)
Surface expression of TLR and leukocyte activation markers													
Neutrophils:	TLR-2	0.559	(<0.001)	-0.381	(0.012)	-0.427	(0.004)			-			
	TLR-4	0.402	(0.007)	0.943	(<0.001)	0.856	(<0.001)	0.523	(<0.001)			-	
Monocytes:	TLR-2	-		0.319	(0.037)			0.679	(<0.001)	0.559	(<0.001)	0.402	(0.007)
	TLR-4	0.319	(0.037)	-		0.891	(<0.001)	0.514	(<0.001)	-0.381	(0.012)	0.943	(<0.001)
	CD36			0.891	(<0.001)	-		0.538	(<0.001)	-0.427	(0.004)	0.856	(<0.001)
	CD86	0.679	(<0.001)	0.514	(<0.001)	0.538	(<0.001)	-				0.523	(<0.001)
Clinical and biochemical variables													
Body mass index				-0.394	(0.008)	-0.455	(0.002)	-0.410	(0.006)			-0.384	(0.010)
Waist				-0.454	(0.002)	-0.477	(0.001)					-0.436	(0.003)
Waist-hip ratio				-0.528	(<0.001)	-0.514	(<0.001)			0.417	(0.005)	-0.456	(0.002)
Total T										0.334	(0.028)	-0.321	(0.034)
Free T				-0.304	(0.045)					0.337	(0.027)	-0.322	(0.033)
E_2													
Free E_2													
Ratio Free T/E_2													
Insulin								-0.361	(0.017)				
Glucose				-0.630	(<0.001)	-0.622	(<0.001)	-0.526	(<0.001)			-0.606	(<0.001)
Insulin sensitivity index				0.394	(0.008)	0.333	(0.027)	0.337	(0.027)			0.354	(0.018)
HOMA-IR								-0.435	(0.004)				

Abbreviations: E_2, estradiol; HOMA-IR, homeostasis model assessment of insulin resistance; T, testosterone. Data were submitted to Pearson's or Spearman´s correlation analysis as needed. Only statistically significant correlations are shown.
